# p600 regulates spindle orientation in apical neural progenitors and contributes to neurogenesis in the developing neocortex

**DOI:** 10.1242/bio.20147807

**Published:** 2014-05-08

**Authors:** Camille Belzil, Naoyuki Asada, Kei-ichiro Ishiguro, Takeo Nakaya, Kari Parsons, Valentina Pendolino, Gernot Neumayer, Marina Mapelli, Yoshihiro Nakatani, Kamon Sanada, Minh Dang Nguyen

**Affiliations:** 1 Hotchkiss Brain Institute, University of Calgary, Departments of Clinical Neurosciences, Cell Biology and Anatomy, and Biochemistry and Molecular Biology, 3330 Hospital Drive NW, Heritage Medical Research Building, Calgary, AB T2N 4N1, Canada; 2 Molecular Genetics Research Laboratory, Graduate School of Science, The University of Tokyo, Hongo 7-3-1, Bunkyo-ku, Tokyo, 113-0033, Japan; 3 Dana Farber Cancer Institute, 44 Binney Street, Smith Building 836, Boston, MA 02115, USA; 4 European Institute of Oncology, Department of Experimental Oncology, Via Adamello, 16-20139 Milan, Italy; 5 Institute of Molecular and Cellular Biosciences, University of Tokyo, 1-1-1 Yayoi, Tokyo 113-0032, Japan

**Keywords:** p600, UBR4, Ndel1, Neurogenesis, Apical neural progenitors, Spindle orientation

## Abstract

Apical neural progenitors (aNPs) drive neurogenesis by means of a program consisting of self-proliferative and neurogenic divisions. The balance between these two manners of division sustains the pool of apical progenitors into late neurogenesis, thereby ensuring their availability to populate the brain with terminal cell types. Using knockout and *in utero* electroporation mouse models, we report a key role for the microtubule-associated protein 600 (p600) in the regulation of spindle orientation in aNPs, a cellular event that has been associated with cell fate and neurogenesis. We find that p600 interacts directly with the neurogenic protein Ndel1 and that aNPs knockout for p600, depleted of p600 by shRNA or expressing a Ndel1-binding p600 fragment all display randomized spindle orientation. Depletion of p600 by shRNA or expression of the Ndel1-binding p600 fragment also results in a decreased number of Pax6-positive aNPs and an increased number of Tbr2-positive basal progenitors destined to become neurons. These Pax6-positive aNPs display a tilted mitotic spindle. In mice wherein *p600* is ablated in progenitors, the production of neurons is significantly impaired and this defect is associated with microcephaly. We propose a working model in which p600 controls spindle orientation in aNPs and discuss its implication for neurogenesis.

## INTRODUCTION

In the developing neocortex, neurogenesis requires the survival, renewal and differentiation of apical neural progenitors (aNPs). Composed of neuroepithelial stem cells (NESCs) and their derivative, the radial glia cells (RGCs), aNPs give rise directly to neurons populating the layers of the cortex or indirectly through the generation of basal progenitors (BPs) in the sub-ventricular zone (Götz and Huttner, 2005; Kriegstein and Alvarez-Buylla, 2009; Rakic et al., 2009; Sessa et al., 2010; Postiglione et al., 2011). During early phases of mammalian corticogenesis, aNPs divide symmetrically to expand the progenitor pool. As corticogenesis proceeds, they then divide asymmetrically to generate either one neuron and one aNP, or one neuron and one BP that will produce two neurons (Götz and Huttner, 2005).

The orientation of the mitotic spindle, perpendicular to the cleavage furrow, is highly linked to the manner of cell division in aNPs (Fietz and Huttner, 2011; Götz and Huttner, 2005; Huttner and Kosodo, 2005; Buchman and Tsai, 2007; Kriegstein and Alvarez-Buylla, 2009; Lancaster and Knoblich, 2012). During the early expansion phase, the spindle is precisely oriented horizontally relative to the apical surface, resulting in a vertical cleavage plane. During the neurogenic phase, the fraction of aNPs with obliquely/vertically-oriented spindle increases (Götz and Huttner, 2005; Kriegstein and Alvarez-Buylla, 2009; Rakic et al., 2009; Sessa et al., 2010; Postiglione et al., 2011). Such plan of division is often associated with an unequal segregation of fate determinant signaling molecules (Par3/Par6/aPKC, numb/numb-like, Neuregulin/APC, Pals1) (Hur and Zhou, 2010; Kim et al., 2010; Petersen et al., 2002; Kim and Walsh, 2007; Bultje et al., 2009; Yokota et al., 2009), the apical/basal membrane domain and/or organelles (primary cilium, centrosome) (Wang et al., 2009), thereby implicating oblique/vertical spindle orientation in asymmetric outcome of daughter cell fates. Though the correlation between spindle orientation and cell fate is demonstrably imperfect and thus not exclusively causal, the close link between spindle orientation, mitotic delay, and severe neurogenic failure warrants study.

The formation and orientation of the mitotic spindle depends on the polymerization, stability and capture of microtubules (MTs) at the plus-end (Wynshaw-Boris et al., 2010). In the neocortex around embryonic day (E) 12, Ndel1 and its homolog Nde1 promote symmetric proliferative division of aNPs. Via association to Lis1 and Dynein, they regulate the formation of aster MTs, their capture at the cell cortex and stabilize the horizontally-aligned spindle (Alkuraya et al., 2011; Pramparo et al., 2011; Feng and Walsh, 2004; Yingling et al., 2008; Moon et al., 2014). Depletion of Ndel1 or Lis1 causes randomization of the spindle orientation, an event that could trigger apoptosis or precocious neuronal differentiation of aNPs, thereby resulting in depletion of progenitor pools and an overall marked decrease in neuronal production (Yingling et al., 2008). Thus, spindle orientation is linked to the proliferation, fate and survival of aNPs.

Recently, studies have shown that p600 (also known as UBR4), a 600 kDa multi-functional protein enriched in the brain, is essential for fetal murine development (Nakatani et al., 2005; Tasaki et al., 2005; Shim et al., 2008; Nakaya et al., 2013). Two mouse strains lacking *p600* (p600^−/−^) were found to be embryonic lethal between E9.5 and E14.5 (depending on the strain, genetic background, and individual variation) with abnormal development of several embryonic tissues (including microcephalic brain) and extra-embryonic organs (yolk sac, placenta) and an overall growth defect (Nakaya et al., 2013; Tasaki et al., 2013). The pleiotropic defects in p600 null mice are consistent with the ubiquitous expression of the protein and its fundamental roles in different cell types. p600's functions encompass protein degradation (through the proteasome or autophagy), cell anchorage, cell survival, cell transformation, calcium signaling and cytoskeletal remodeling (DeMasi et al., 2005; Huh et al., 2005; Nakatani et al., 2005; Tasaki et al., 2005; Shim et al., 2008; Belzil et al., 2013).

In the brain, p600 has been studied as a MT-associated protein during neuronal migration and as Calmodulin-binding partner for the survival of active cultured hippocampal neurons (Belzil et al., 2013; Shim et al., 2008). Using *in utero* electroporation of shRNA, we initially found that p600-depleted neurons were positioned aberrantly in the developing cortex. The phenotype was attributed to a neuronal migration defect and at the cellular level, to the crooked, thin and zigzag leading process caused by loss of the MT stabilizing function of p600 (Shim et al., 2008). However, the brain phenotype of p600 knockout mice appears around the onset of neurogenesis (Nakaya et al., 2013). We therefore reasoned that the migration defect could not fully account for the brain deformities, and instead suspected defects in neural progenitor populations. Based on these findings, we hypothesized that p600 is expressed in mitotic NPs and, by virtue of its MT-associated protein function, affects MT spindle orientation in NPs to potentially impact neurogenesis. To test this hypothesis, we used mice with a targeted disruption of *p600* in epiblasts, i.e. pluripotent epithelial stem cells including aNPs (p600^SC−/−^, see Materials and Methods and Nakaya et al., 2013) combined with *in utero* electroporation of p600 shRNAs. p600^SC−/−^ animals die variably between E12.5 and E14.5 (Nakaya et al., 2013), thereby providing a short time window to study aNPs.

## MATERIALS AND METHODS

### Generation of p600^SC−/−^ animals

p600^SC−/−^ were generated by crossing the p600 lox allele with the Sox2-Cre transgenic mice (Nakaya et al., 2013). Briefly, Sox2-Cre male transgenic mice with the p600^LoxP/WT^ allele were bred with female p600^LoxP/LoxP^ animals to generate embryos with 4 genotypes: Sox2-Cre+; p600^KO/KO^ (or p600^SC−/−^), Sox2-Cre−; p600^KO/LoxP^ (or p600^LoxP/−^); Sox2-Cre+; p600^KO/WT^ (or p600^SC+/−^), Sox2-Cre−; p600^WT/LoxP^ (or p600^LoxP/+^). The *Sox2* promoter is active in epithelial cell lineage/epiblast including the neuroepithelium by E6.5 (Hayashi et al., 2002; Ellis et al., 2004; Bani-Yaghoub et al., 2006), and was therefore guaranteed to drive p600 ablation in the earliest populations of neural progenitors in the brain. This strategy not only avoids the early mortality associated with extra-embryonic tissue defects (i.e. placenta and yolk sac) (Nakaya et al., 2013; Tasaki et al., 2013) but also precludes an involvement of the placenta in any potential brain phenotype (Hayashi et al., 2002). Genotypes for p600^SC−/−^ mice were assayed by PCR. The mice were housed and handled according to Canadian Council on Animal Care guidelines and experimentation approved by the Health Sciences Animal Care Committee.

### Western blot, cloning, transfection and immunoprecipitations

#### Western blot

Total protein extracts of mouse embryos and HeLa cells were obtained by homogenization in SDS-urea (0.5% SDS, 8 M urea in pH 7.4 phosphate buffer) or Triton X-100 (10 mM Tris-HCl (pH 7.5), 150 mM NaCl, 1 mM EDTA (pH 8.0) and 1% Triton X-100) buffer. The protein concentration was estimated by the Bradford or DC assay (Bio-Rad Laboratories, Hercules, CA). Proteins were fractionated by SDS-PAGE and blotted on a nitrocellulose or PVDF membrane for Western blot analysis. Membranes were incubated with antibodies (Abs) specific against p600, Ndel1, Lis1 (all three Abs are home-made) and GFP (B-2, Santa Cruz). The Western blots were examined using a chemiluminescence kit from NEN Life Science (Boston, MA). Quantitations were corrected with levels of actin, α-tubulin and FAK and performed with the Labscan program (Image Master, 2D software v 3.10, Amersham Pharmacia Biotech).

#### Cloning

p600^3214–3899^, p600^3910–4851^ and p600^4480–5183^ were amplified from a human fetal cDNA library and cloned into pcDNA 3.1 (+) (Invitrogen) with an N-terminal FLAG tag. Expression was verified by Western blotting directed against the FLAG epitope.

#### Cell culture and transfection

HeLa cells were cultured in DMEM supplemented with 10% FBS and 1× penicillin/streptomycin (GIBCO). Upon reaching 70% confluence they were transfected with the truncated fragments of p600 using Lipofectamine 2000 (Invitrogen, Carlsbad, CA) according to manufacturer protocols.

#### Immunoprecipitations

Immunoprecipitations were performed as described previously (Nguyen et al., 2004; Shu et al., 2004; Sanada and Tsai, 2005).

### Immunohistochemistry/immunofluorescence and spindle orientation calculation

For immunohistochemistry, mice were anesthetized with avertin and intracardially perfused with PBS, followed by 4% paraformaldehyde. Brains were cryo-protected or mounted on paraffin prior to sectioning into 6 µm thick paraffin coronal slices. The sections were then deparaffinized, rehydrated, stained with H&E or processed for DAB immunohistochemistry. In the latter case, antigen retrieval was performed by microwave irradiation and/or 88% formic acid treatment. Sections were then incubated with Tuj-1 primary antibodies (1:1000; Sigma) overnight at 4°C. Bound antibodies were detected by standard streptavidin-biotin-peroxidase methods (Vector Laboratories, Burlingame, CA). The orientation of cleavage was calculated based on the alignment of chromosomes stained with H&E as described previously (Sanada and Tsai, 2005).

For immunofluorescence staining, embryonic brains were fixed with 4% paraformaldehyde in PBS for 30 minutes at room temperature and cryoprotected in 25% sucrose in PBS overnight at 4°C. Thereafter, the brains were embedded in a solution of a 2:1 mixture of 25% sucrose/PBS and OCT compound (Sakura), frozen by liquid nitrogen, and stored at −80°C until use. Thick cryosections (20 µm) were made. The brain sections were pre-treated with a HistoVT One solution (Nakalai Tesque) at 70°C for 15 min, incubated with blocking solution [3% (w/v) BSA, 5% (v/v) FBS, and 0.3% (w/v) Triton X-100 in PBS] and then incubated with primary antibodies overnight at 4°C. Primary antibodies used were mouse anti-N-Cadherin (1:200; BD Transduction), rabbit anti-phospho-histone H3 (1:1000; Millipore), rabbit anti-Tbr2 (1:500; Chemicon) and rabbit anti-Pax6 (1:500; Chemicon). The sections were then incubated with Alexa488/Cy3/Cy5/Alexa649-conjugated secondary antibodies overnight at 4°C and mounted in a Prolong Gold mounting solution (Invitrogen). Nuclei were stained with 4′,6′-diamidino-2-phenylindole (DAPI) or TO-PRO-3 iodide (Invitrogen). Images were obtained with a Carl Zeiss LSM510 confocal microscope. The orientation of cleavage was calculated based on the alignment of chromosomes stained with DAPI or TO-PRO-3 iodide as described previously (Sanada and Tsai, 2005).

### *In utero* electroporation

The two specific and published RNA interference (RNAi) sequences for p600 are base pairs GCAGTACGAGCCGTTCTAC and AATGATGAGCAGTCATCTA (Shim et al., 2008). A random sequence without homology to any known mRNA was used for control RNAi. The p600^4480–5183^ construct was generated as described below. An empty vector was used as a control. All RNAi and cDNA constructs were previously tested in cell lines and primary neuronal cultures by both Western blots and immunofluorescence staining. *In utero* electroporation was performed at E13 as described previously (Sanada and Tsai, 2005; Shim et al., 2008) and neocortices were analyzed at E14 or E15. In brief, DNA solution (1–2 µl) in PBS containing 0.01% fast green was injected into the lateral ventricle of E13 mouse embryos. Thereafter, electroporation (39–42 V, five 50 milliseconds square pulse with 950 milliseconds intervals; CUY21-EDIT, Nepa gene, Chiba, Japan) was carried out. All animal experiments were conducted in accordance with guidelines set by The University of Tokyo and approved (permit number 21-01) by the Committee on Animal Care and Use of the Graduate School of Science in The University of Tokyo.

### Biochemical analyses

The region of human p600 encompassing residues 4480–5183 (referred to as p600^4480–5183^) was expressed as GST-fusion from pGEX-6PI (GE-Healthcare) at 20°C in *E. coli* strain BL21-Rosetta for 12 h after induction with 0.1 mM IPTG. Cell were lysed by sonication in 0.1 M Tris-HCl pH 8, 0.3 M NaCl, 10% glycerol, 0.5 mM EDTA, 1 mM DTT and protease inhibitor cocktail Set III (Merck–Millipore). After clearing by ultracentrifugation, the lysate was applied to glutathione sepharose beads equilibrated in lysis buffer and incubated for 2 h. Beads were washed with 30 volumes of lysis buffer and equilibrated in cleavage buffer (20 mM Tris-HCl pH 8, 40 mM NaCl, 5% glycerol, 0.5 mM EDTA and 1 mM DTT). To remove the GST tag from p600^4480–5183^, 10 units of PreScission protease (GE-Healthcare) per mg of substrate were added, and the mixture was incubated for 16 h at 4°C. The cleaved product was applied to a 6-ml Resource Q column (GE-Healthcare), eluted with a NaCl gradient, and concentrated with Vivaspin concentrators. The entire purification was carried out at 4°C. Ndel1^1–201^ was expressed with an N-terminal hexahistidine-tag from a pETM-14 vector in BL21-Rosetta cells. Clear lysates in 0.1 mM Tris-HCl buffer (pH 8), 0.3 M NaCl, 5 mM imidazole, 2 mM 2-mercaptoethanol and protease inhibitors were applied to Ni-NTA agarose beads (QIAGEN) and eluted with 0.1 M imidazole. The sample buffer was then desalted, and the construct was further purified by ion exchange as described for p600^4480–5183^. Purified proteins were stored at −80°C in aliquots.

Analytical size-exclusion chromatography experiments were performed at 4°C on a Superdex 200 5/150 column equilibrated in 10 mM HEPES buffer (pH 7.5), 0.15 M NaCl, and 1 mM DTT. Before injection, purified p600^4480–5183^ and Ndel1^1–201^ were mixed at 50 µM concentration and incubated for 30 min on ice. Eluted fractions were collected and analyzed by SDS-PAGE.

## RESULTS

### Apical neural progenitors of the developing neocortex express p600

**Fig. 1.**
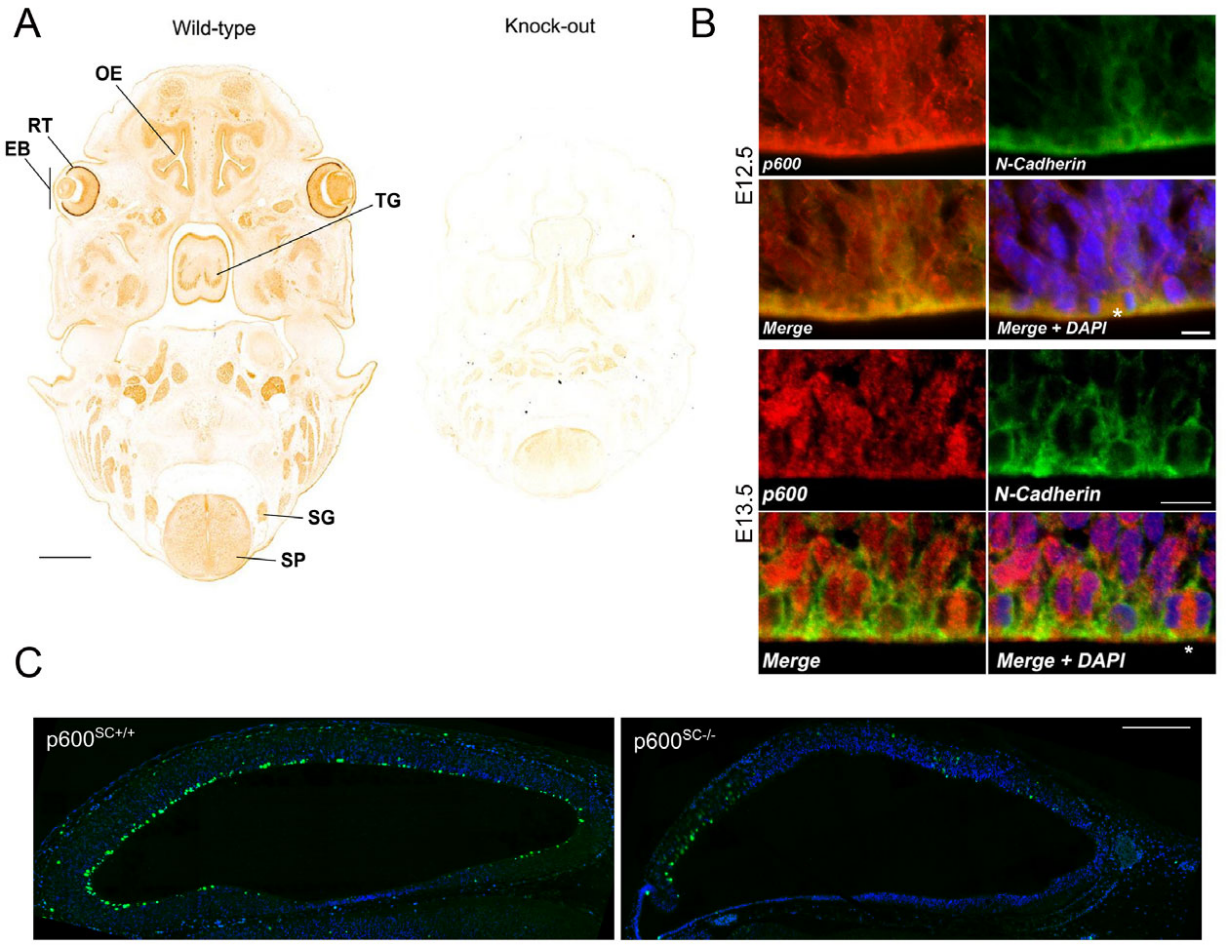
p600 is expressed in apical neural progenitors at E12.5–13.5 and reduced number of apical neural progenitors in E12.5 p600^SC−/−^ brains. (A) Immunohistochemistry of p600 in the developing E13.5 WT embryos reveals p600 expression in the eye bud (EB), particularly in the retina (RT), in the olfactory epithelium (OE), the tongue (TG), the spine (SP) and the spinal ganglia (SG). Specificity of the signals was confirmed with tissues from p600^SC−/−^ animals (right). (B) p600 is strongly expressed in aNPs of the ventricular zone in the neocortex at E12.5 and E13.5 as revealed by co-labeling with N-Cadherin antibody. Note the expression of p600 in mitotic cells or cells finishing mitosis (asterisk). (C) Reduced levels of phosphorylated histone H3 in the ventricular zone of the neocortex of p600^SC−/−^ mice at E12.5. Scale bars: 500 µm (A), 12 µm (B), 200 µm (C).

p600 is expressed in embryonic brain neurons (Shim et al., 2008). To further examine the expression pattern of p600 during brain development and to test our homemade p600 antibodies on embryonic tissues, we performed immunochemical DAB staining on sections of wild-type E13.5 embryo. As shown in Fig. 1A, p600 is expressed in many embryonic tissues, including the spinal cord, and olfactory epithelium. The specificity of the homemade antibodies was demonstrated previously using cells depleted of p600 by RNAi (Shim et al., 2008) and further confirmed with sections derived from age-matched sibling p600^−/−^ animals (Fig. 1A). Using the same antibodies, we found p600 to be expressed in the embryonic neocortex. In particular, p600 is expressed in aNPs of the ventricular zone at embryonic days 12.5 and 13.5 (Fig. 1B; supplementary material Fig. S1), as revealed by co-labeling with N-Cadherin antibody. These results indicate that p600 is expressed in neurogenic proliferative regions of the developing neocortex.

### Reduced number of apical neural progenitors in p600^SC−/−^ brains

To determine whether aNPs are affected in p600^SC−/−^ animals, immunofluorescent staining with the mitotic marker histone H3 phosphorylated at Ser10 (PH3) was performed at E12.5 followed by confocal microscopy analysis. In control p600^SC+/−^ animals, small patches of intense PH3 signals were found in the ventricular and sub-ventricular zones (Fig. 1C). The strongest signals lining the ventricular zone mark mitotic aNPs i.e. mitotic NESCs and RGCs. In contrast, in p600^SC−/−^ animals these immunoreactivities were strikingly scarce. This result suggests that by E12.5, mitotic aNPs are depleted in p600^SC−/−^ mice.

### Randomization of spindle orientation in aNPs of p600^SC−/−^ neocortex and p600 RNAi-electroporated neocortices

**Fig. 2.**
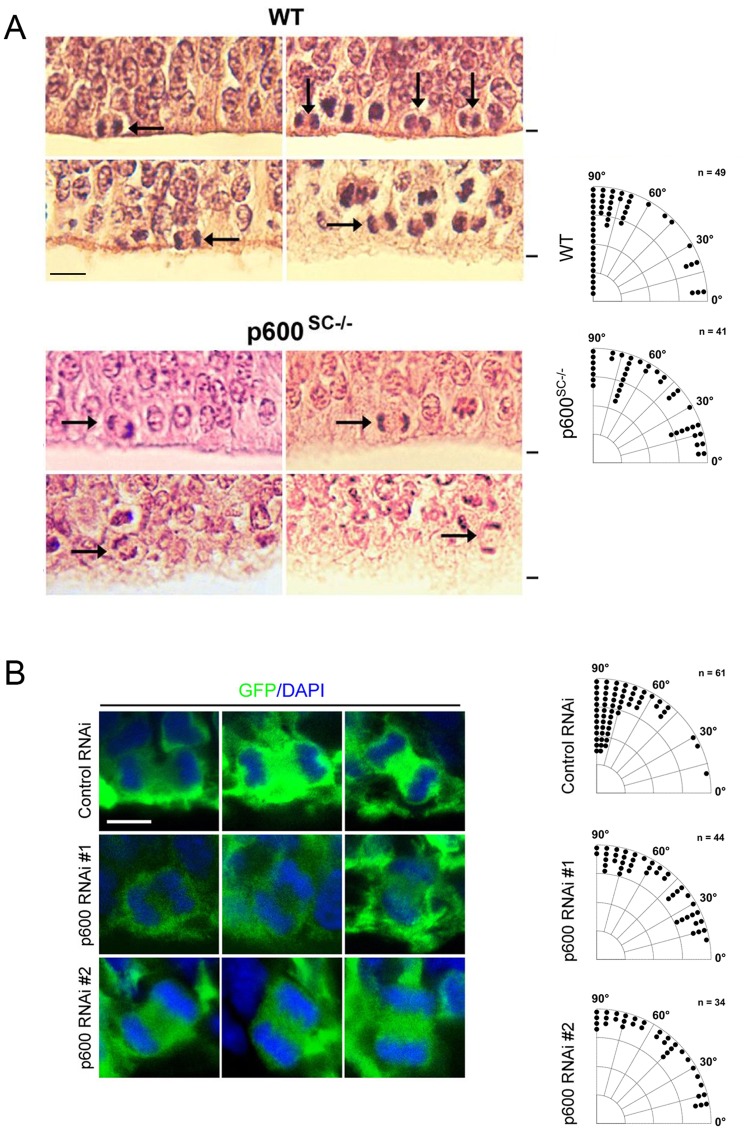
p600 is required for the orientation of the mitotic spindle in E12.5 and E15 apical neural progenitors. (A) p600 is required for vertical spindle orientation in NESCs. Angle histograms showing the relative frequencies of spindle orientations in increments of 5°. At E12.5 the mitotic spindle is tilted by 28% in aNPs (mostly NESCs in the caudal region) of p600^SC−/−^ neocortices [control p600^SC+/−^: 71.6±26.7° (mean ± SD), n = 49 cells; p600^SC−/−^: 52.7±28.6°, n = 40 cells; U = 580, p<0.0005 *vs* control by one-tailed Mann–Whitney U test]. Representative photographs of the ventricular zones of WT and p600^SC−/−^ neocortices at E12.5 stained with H&E are shown. Arrows point to examples of condensed chromosomes in dividing aNPs lining the ventricle. The black stripe indicates the edge of the ventricle. (B) p600 is required for vertical spindle orientation in RGCs. Angle histograms showing the relative frequencies of spindle orientations in increments of 5°. The spindle orientation of p600 RNAi-electroporated neural progenitors at E15 (mostly RGCs) deviates by ∼29% when compared to control RNAi-treated cells [control: 73.1±16.9° (mean ± SD), n = 61 cells; RNAi #1: 54.2±25.5°, n = 44 cells; U = 644, p<0.000005; RNAi #2: 52.3±26.9, n = 34 cells; U = 555, p<0.0001 *vs* control by one-tailed Mann–Whitney U test]. Representative confocal micrographs of RGCs lining the ventricle of control RNAi/GFP, p600 RNAi #1/GFP or p600 RNAi #2/GFP co-electroporated neocortices at E15 are shown. Condensed chromosomes were labeled with DAPI. *In utero* electroporation was performed at E13 and neocortices were analyzed at E15. Scale bars: 12 µm.

Dysregulation of one of several cellular processes controlled by p600 (such as cell survival, autophagy, cell adhesion, cell differentiation and apoptosis) could account for the depletion of aNPs in p600^SC−/−^ neocortex. By virtue of its MT-associated protein function, we sought to examine the orientation of the mitotic spindle in aNPs depleted of p600. Spindle orientation has been proposed to affect self-proliferative and neurogenic divisions of aNPs (Yingling et al., 2008; Sessa et al., 2010; Postiglione et al., 2011). By no means does this selective analysis exclude the possibility that other functions of p600 are compromised in aNPs of p600^SC−/−^ mice (see Discussion). We first analyzed the cleavage plane of aNPs from p600^SC−/−^ animals. In aNPs lining the ventricular zone of E12.5 wild-type control and p600^SC−/−^ brains, which are mainly NESCs in the caudal region (Sahara and O'Leary, 2009), the orientation of cleavage was calculated based on the angle between the spindle orientation (i.e. alignment of chromosomes stained with H&E) (Fig. 2A) and the ventricular surface. The analysis revealed that the spindle orientation of aNPs derived from p600^SC−/−^ neocortices deviates by ∼28% from the largely vertical cleavage planes of WT progenitors (data presented as mean ± SD: control p600^SC+/−^: 71.6±26.7°, n = 49 cells; p600^SC−/−^: 52.7±28.6°, n = 40 cells; U = 580, p<0.0005 *vs* control by one-tailed Mann–Whitney U test) (see Fig. 2A for histograms and representative cells). In sum, NESCs in p600^SC−/−^ brains have tilted mitotic spindle.

We also determined the spindle orientation in RGCs electroporated with control or p600 RNAis at E13 and analyzed at E15. E13 is an active neurogenic phase, and the majority of aNPs are RGCs at this stage. The calculation was made based on the orientation of condensed chromatin labeled with DAPI as described above (see Fig. 2B for histograms and representative cells). The analysis revealed that the spindle orientation of p600 RNAi-electroporated RGCs deviates by ∼29% from the mainly vertical cleavage planes of control cells (data presented as mean ± SD: control: 73.1±16.9°, n = 61 cells; RNAi #1: 54.2±25.5°, n = 44 cells; U = 644, p<0.000005; RNAi #2: 52.3±26.9, n = 34 cells; U = 555, p<0.0001 by one-tailed Mann–Whitney U test) (Fig. 2B). Thus, RGCs in p600 RNAi-electroporated neocortices also display randomized spindle orientation. In sum, both aNPs of p600^SC−/−^ neocortex and p600 RNAi-electroporated neocortices display randomization of spindle orientation.

### p600 interacts with the neurogenic protein Ndel1

**Fig. 3.**
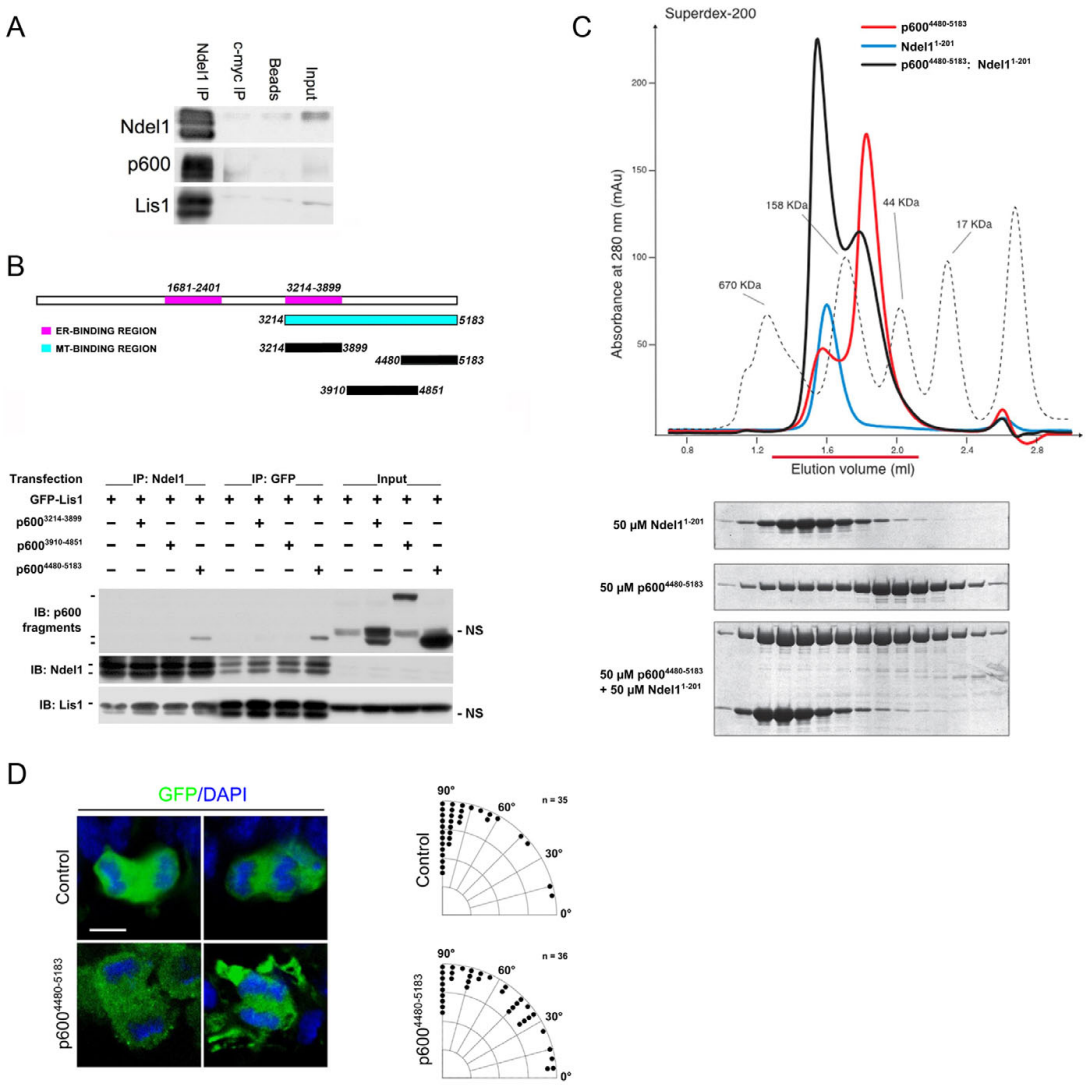
p600 interacts directly with Ndel1. (A) Using Ndel1 antibodies, p600 and Lis1 co-immunoprecipitate with Ndel1 from mouse brain lysates. Myc antibodies and beads alone were used as negative controls. (B) A C-terminal FLAG-tagged fragment of p600 containing residues 4480–5183 expressed ectopically in HeLa cells co-immunoprecipitates with endogenous Ndel1 and ectopic GFP-Lis1. p600 fragments containing residues 3214–3899 and 3910–4851 did not co-immunoprecipitate with endogenous Ndel1 and ectopic GFP-Lis1. (C) The Ndel1 coiled-coil domain (a.a. 1–201; blue trace) elutes at an apparent molecular weight of about 180 kDa, earlier than expected for its molecular weight due to the elongated shape of the coiled-coil. p600^4480–5183^ (red trace) elutes mostly as a monomeric species between the 158 kDa and the 44 kDa markers. When combined stoichiometrically at a 50 µM concentration, the two proteins form a complex eluting before the 158 kDa marker. For each run, the content of fifteen fractions was analyzed by SDS-PAGE followed by Coomassie staining. (D) The Ndel1/p600 interaction is required for vertical spindle orientation in RGCs. Angle histograms showing the relative frequencies of spindle orientations in increments of 5°. The spindle orientation of p600^4480–5183^-expressing aNPs deviates by ∼29% when compared to control cells [control: 75.2±20.4° (mean ± SD), n = 35 cells; p600^4480–5183^: 58.4±28.1°, n = 36 cells; U = 421, p = 0.008 by one-tailed Mann–Whitney U-test]. Representative confocal pictures of aNPs (most likely RGCs) lining the ventricular zone of control (empty vector)/GFP or p600^4480–5183^/GFP co-electroporated neocortices at E15 are shown. Condensed chromosomes were labeled with DAPI. *In utero* electroporation was performed at E13 and neocortices were analyzed at E15. Scale bar: 5 µm.

The randomization of spindle orientation observed in NPs of p600^SC−/−^ animals are reminiscent of those exhibited by mice lacking Ndel1, its homolog Nde1, Lis1, or with altered Ndel1/Lis1/Dynein function (Alkuraya et al., 2011; Yingling et al., 2008; Pramparo et al., 2011). These similar phenotypes suggested the possibility that p600 may associate with the Ndel1/Lis1/Dynein complex. To determine whether p600 associates with Ndel1 and Lis1, we first performed co-immunoprecipitation experiments. Using Ndel1 antibodies, p600 and Lis1 were co-immunoprecipitated with Ndel1 from mouse brain lysates (Fig. 3A). As a microtubule-associated protein, p600 exhibits at least one MT-binding domain located in the C-terminus of the protein (a.a. 3214–5183) (Shim et al., 2008) (Fig. 2B). Since Ndel1 and Lis1 also act on MTs we hypothesized that the C-terminus of p600 served as a binding interface for Ndel1 and/or Lis1. Therefore, we performed co-immunoprecipitations from HeLa cell extracts expressing GFP-Lis1 and one of the three C-terminal fragments of p600 (a.a. 3214–3899; a.a. 3910–4851; a.a. 4480–5183) comprising the MT-binding region. We found that p600^4480–5183^, but not p600^3214–3899^ or p600^3910–4851^, was co-immunoprecipitated with endogenous Ndel1 (Fig. 3B). As expected, GFP-Lis1 was also immunoprecipitated with Ndel1 (Fig. 3B).

Based on phenotypic similarities between p600-depleted and Ndel1/Nde1-depleted neocortices (Alkuraya et al., 2011; Feng and Walsh, 2004; Shu et al., 2006; Shim et al., 2008; Yingling et al., 2008; Pramparo et al., 2011; and this study), we next determined whether p600^4480–5183^ and Ndel1 interact directly in a complex in solution. To this aim, we performed size-exclusion chromatography (SEC) experiments with recombinant p600^4480–5183^ and the coiled-coil region of Ndel1 (residues 1–201), hereafter referred to as Ndel1^1–201^. The elution volume of Ndel1^1–201^ in isolation (Fig. 3C, blue trace) is compatible with either a monomer with elongated shape or an oligomer. To distinguish between the two possibilities, we performed Static Light Scattering analysis of the same construct, which revealed that Ndel1^1–201^ is dimeric in solution (data not included; Derewenda et al., 2007). The elution profile of p600^4480–5183^ presents two peaks (red trace). The major peak is between the 44 kDa and the 158 kDa markers, and is consistent with a monomer (∼75 kDa), while the other peak elutes earlier than the 158 kDa marker thus suggesting that at 50 µM a minor proportion of p600^4480–5183^ is dimeric. When we combined stoichiometric amounts of p600^4480–5183^ and Ndel1^1–201^ at a 50 µM concentration, the peak corresponding to the monomeric pool of p600^4480–5183^ decreased (black trace), and Ndel1^1–201^ eluted at higher molecular weight than Ndel1^1–201^ in isolation. This result indicates that p600^4480–5183^ associates directly with Ndel1^1–201^. When the binding was performed at 10 µM, p600^4480–5183^ did not enter a complex with Ndel1^1–201^ (data not included), thus suggesting that the interaction is rather weak, likely with a dissociation constant in the order of 30–50 µM. Smaller portions of p600^4480–5183^ were mostly insoluble, precluding further SEC experiments. Only the p600 fragment encompassing residues 4949–5183 was sufficiently soluble for SEC analyses, which revealed that it did not interact with Ndel1^1–201^ (supplementary material Fig. S2). In summary, the C-terminal portion of p600 binds directly to Ndel1 with a low affinity, likely between residues 4480 and 4949.

### Randomization of spindle orientation in neocortices expressing p600^4480–5183^

Based on the interaction between Ndel1^1–201^ and p600^4480–5183^, we determined the orientation of the mitotic spindle in aNPs expressing p600^4480–5183^ at E15. The analysis revealed that the spindle orientation of p600^4480–5183^-electroporated aNPs (mostly composed of RGCs at E15) deviates by ∼29% when compared to control cells (control: 75.2±20.4°, n = 35 cells; p600^4480–5183^: 58.4±28.1°, n = 36 cells; mean ± SD.; U = 421, p = 0.008 by one-tailed Mann–Whitney U-test) (Fig. 3D). These data indicate that disruption of the Ndel1/p600 interaction, like depletion of p600 by shRNA or gene knockout, randomizes spindle orientation in aNPs.

### Decreased number of Pax6-positive aNPs and increased number of Tbr2-positive BPs in neocortices electroporated with p600 shRNA or p600^4480–5183^

**Fig. 4.**
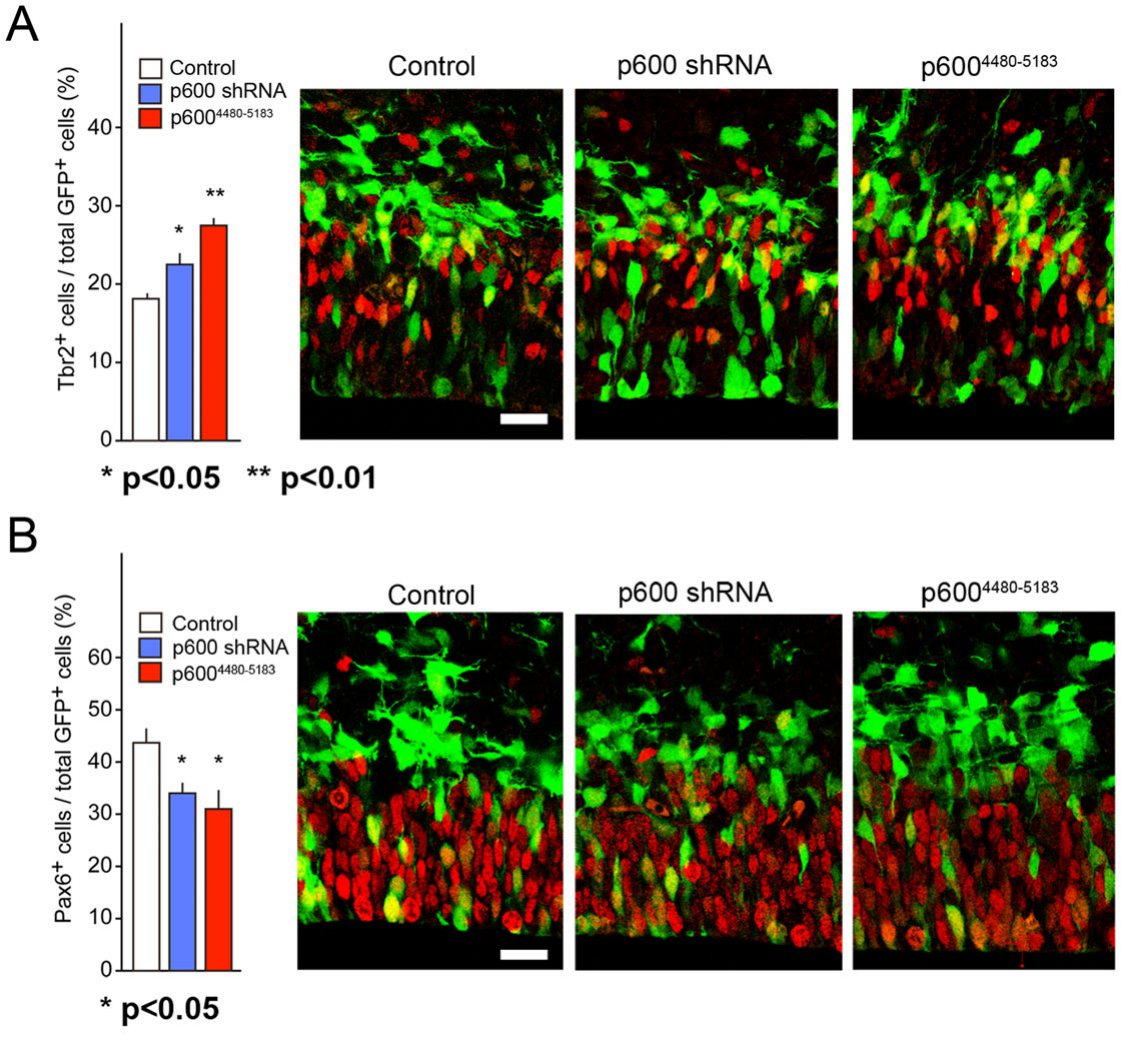
Increased number of Tbr2-positive basal progenitors and decreased number of Pax6-positive apical progenitors in neocortices electroporated with p600 shRNA or p600^4480–5183^. (A) The fraction of Tbr2-positive basal progenitors over total GFP-positive cells is greater in p600 shRNA and p600^4480–5183^ electroporated neocortices than in those electroporated with control shRNA. [control: 18.1±0.67%; RNAi #1: 22.5±1.5%; p600^4480–5183^: 27.4±0.95% (mean ± SEM); n = 4 (control, 1004 cells), 4 (p600 RNAi #1, 688 cells); p<0.05 by two-tailed Student's t-test; 4 (p600^4480–5183^, 693 cells); p<0.01 by two-tailed Student's t-test]. Green (GFP), Red (Tbr2). (B) The fraction of Pax6-positive apical progenitors is reduced in p600 shRNA and p600^4480–5183^ electroporated neocortices than in those electroporated with control shRNA. Note the inverted proportion of basal *vs* apical progenitors in p600 shRNA and p600^4480–5183^ electroporated neocortices. [control: 43.6±2.7%; RNAi #1: 34.0±2.1%; p600^4480–5183^: 31.0±3.4% (mean ± SEM); n = 3 (control, 446 cells), 3 (p600 RNAi #1, 519 cells); p<0.05 by two-tailed Student's t-test; 3 (p600^4480–5183^, 258 cells); p<0.05 by two-tailed Student's t-test]. Scale bars: 20 µm; Green (GFP), Red (Pax6).

Ndel1 and its close homolog Nde1 are important for proliferative/neurogenic divisions and cell fate (Alkuraya et al., 2011; Feng and Walsh, 2004; Yingling et al., 2008; Pramparo et al., 2011). We hypothesized that if p600 was acting through Ndel1, then disruption of p600 or its interaction with Ndel1 ought to alter cell fate. Thus, at E13 we electroporated aNPs with p600 RNAi, or with p600^4480–5183^, in order to uncouple the interaction between Ndel1 and endogenous p600. For this, vector encoding the specific RNAi #1 for p600 (Shim et al., 2008; Belzil et al., 2013), or a vector encoding p600^4480–5183^ was co-electroporated with GFP vector in E13 mouse neocortices. Interestingly, we found that the number of Tbr2-positive cells over the total number of GFP-positive cells at E14 were increased in p600 shRNA and, particularly, in p600^4480–5183^-expressing neocortices (Fig. 4A). The milder effect of p600 shRNA construct on Tbr2-population (p600 shRNA: 27.5±0.95 versus p600^4480–5183^: 22.5±1.54; p = 0.033 by Student's t-test) may be due to residual levels of p600 in p600 shRNA-introduced cells. Tbr2 is a transcription factor expressed in BP cells that are specified to the neuronal lineage and generate two neurons after cell division (Miyata et al., 2004; Noctor et al., 2004; Sessa et al., 2010). These data show accelerated neuronal differentiation of the progenitors upon expression of p600 shRNA or p600^4480–5183^. In agreement with this view, we found that the number of Pax6-positive cells over the total number of GFP-positive cells were reciprocally decreased in both p600 shRNA and p600^4480–5183^-expressing neocortices (Fig. 4B), indicating depletion of aNPs. Taken together, these results indicate that expression of p600 shRNA or p600^4480–5183^ favors the production of Tbr2-positive BPs at the cost of Pax6-positive aNPs.

### Randomization of spindle orientation in Pax6-positive aNPs expressing p600 shRNA or p600^4480–5183^

**Fig. 5.**
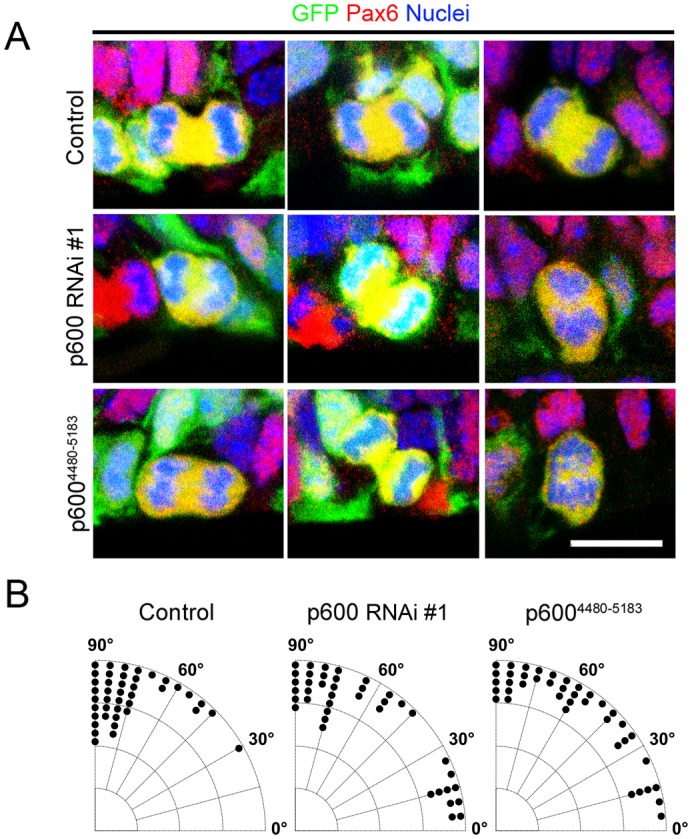
Randomization of spindle orientation in E14 Pax6-positive apical neural progenitors. (A) Representative confocal microscopy pictures of E14 Pax6-positive apical neural progenitors electroporated at E13 with control RNAi, p600 RNAi #1 or a Ndel1-binding p600 fragment comprising residues 4480–5183 (p600^4480–5183^). (B) p600 is required for vertical spindle orientation in Pax6-positive aNPs at E14. Pie charts showing the distribution of Pax6-positive aNPs expressing either a control shRNA, p600 RNAi #1 or p600^4480–5183^ with a spindle orientation of 0–15 (horizontal axe of cleavage), 15–30, 30–45, 45–60, 60–75 or 75–90 (vertical axe of cleavage) degrees. At E14 the mitotic spindle is tilted by ∼28% in Pax6-positive aNPs expressing p600 RNAi #1 or p600^4480–5183^ when compared to control Pax6-positive shRNA-electroporated aNPs (data presented as mean ± SD: control: 75.1±14.4°, n = 41 cells; p600 RNAi #1: 57.9±29.5°, n = 40 cells; p600^4480–5183^: 56.6±26.4°, n = 40 cells; p = 0.008 and p = 0.001 by one-tailed Mann–Whitney U-test, respectively). Scale bar: 10 µm.

We also analyzed at E14 the spindle orientation in RGCs electroporated at E13 with control, p600 RNAi #1 (Shim et al., 2008; Belzil et al., 2013) or p600^4480–5183^. E13–E14 is an active neurogenic phase, and the majority of Pax6-positive aNPs are RGCs at this stage. The calculation was made based on the orientation of condensed chromatin labeled with DAPI as described above (Fig. 2B). The analysis revealed that the spindle orientation of p600 RNAi #1-electroporated aNPs and p600^4480–5183^-electroporated aNPs at E14 deviates by ∼28% when compared to control cells (data presented as mean ± SD: control: 75.1±14.4°, n = 41 cells; p600 RNAi #1: 57.9±29.5°, n = 40 cells; p600^4480–5183^: 56.6±26.4°, n = 40 cells; p = 0.008 and p = 0.001 by one-tailed Mann–Whitney U-test, respectively) (Fig. 5A,B). These data indicate that depletion of p600 by shRNA or disruption of the Ndel1/p600 in Pax6-positive aNPs, like p600 gene knockout in aNPs at E12.5 (Fig. 2), randomizes spindle orientation. This tilted spindle phenotype is circumstantially linked to the depletion of Pax6-positive aNPs (Fig. 4; see Discussion).

### Decreased number of Tuj-1-positive neurons in p600^SC−/−^ microcephalic brains

**Fig. 6.**
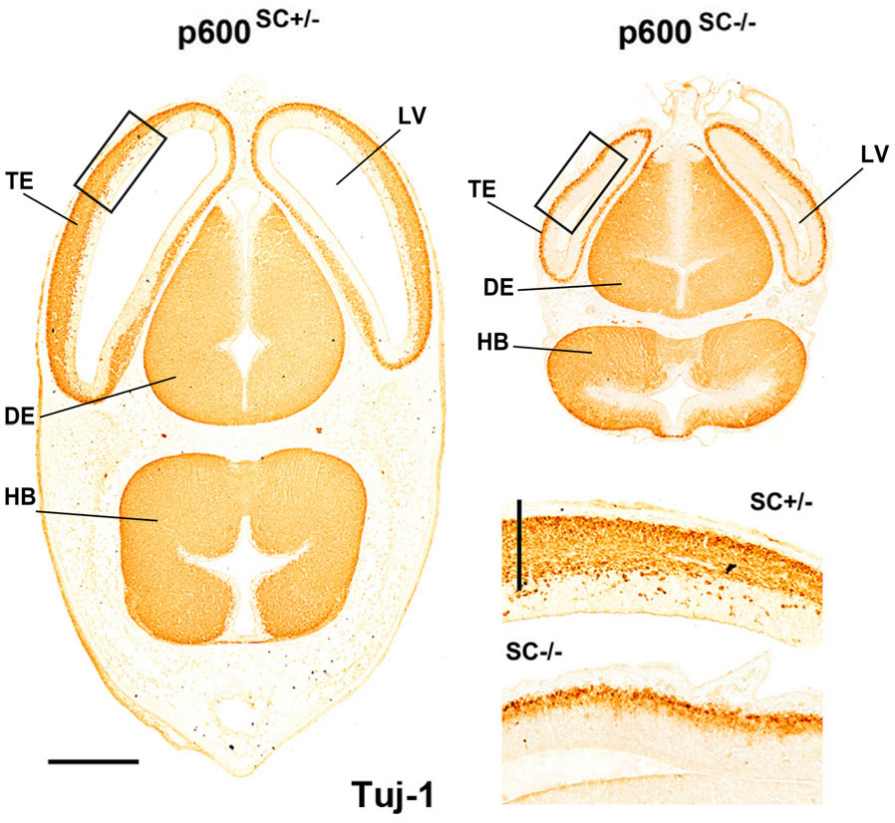
Decreased number of Tuj-1-positive neurons in p600^SC−/−^ brain. Tuj-1 staining for newly-born neurons in E13.5 wild-type brain transverse sections shows expression in the telencephalon (TE), diencephalon (DE) and hindbrain (HB). Zoom in of the insets (black box) illustrates a drastic thinning of the cortical plate in the telencephalon of p600^SC−/−^ mice populated with fewer Tuj-1-positive neurons when compared to p600^SC+/−^. Scale bars: 500 µm, 40 µm (inset).

Following our observations that p600 impacts the abundance of mitotic NPs (Fig. 1C), spindle orientation (Fig. 2B, Fig. 3D, Fig. 5), production of Tbr2-positive BPs at the cost of Pax6-positive aNPs (Fig. 4), we sought to determine whether neurogenesis was altered by assessing the proportion of newly-born neurons with the marker Tuj-1. At E12.5 there was a marked reduction in Tuj-1 immunoreactivity in p600^SC−/−^ microcephalic brains compared to control p600^SC+/−^ littermates (Fig. 6). These results suggest decreased neurogenesis in p600^SC−/−^ brains.

## DISCUSSION

Using mice with disruption of *p600* in epithelial cell lineages and *in utero* electroporation of p600 shRNA and p600 cDNA-encoding plasmids in the neocortex, we discovered that p600 is important for mitotic spindle orientation in aNPs (Fig. 2, Fig. 3D, Fig. 5). This finding is consistent with the cytoskeletal nature of the protein that contains at least two MT-associated protein domains located at the C-terminus (Shim et al., 2008), and with our data showing that this region of p600 interacts directly with the neurogenic Ndel1 protein (Fig. 3) known to regulate spindle orientation. p600 also affects the differentiation of Pax6-positive aNPs into Tbr2-positive BPs destined to become neurons (Fig. 4), spindle orientation in these Pax6-positive aNPs (Fig. 5) and p600^SC−/−^ mice have reduced production of Tuj-1-positive neurons (Fig. 6). It remains unclear whether the neurogenic defects observed in p600^SC−/−^ mice and neocortices depleted of p600 by shRNA or overexpressing p600^4480–5183^ are caused by loss of control of the mitotic spindle orientation. As in other studies, the relationship between altered mitotic spindle orientation and alteration in cell fate could range from partially causal to merely epiphenomenal. By virtue of its MT-associated function in the developing brain (Shim et al., 2008), the idea that p600 is controlling cell fate and neurogenesis via the mitotic spindle is a tempting hypothesis that would required further work to fully test it.

### How does p600 regulate spindle orientation together with Ndel1/Lis1/Dynein?

The randomized spindle orientation of aNPs and abnormal neurogenesis observed in p600^SC−/−^ animals and p600-depleted neocortices (Figs 2, 3, 5) are also observed in aNPs and neocortices of mice with a targeted disruption of either *Lis1*, *Nde1* or *Ndel1* (Alkuraya et al., 2011; Feng and Walsh, 2004; Pramparo et al., 2011; Yingling et al., 2008), suggesting that p600 works in concert with these proteins. We found that the C-terminal portion of p600 (p600^4480–5183^) interacts directly with the N-terminal coiled-coil domain of Ndel1 (Fig. 3), to which Lis1 also binds. *In utero* electroporation of p600^4480–5183^ also randomized spindle orientation in the same way as p600 knockout or depletion (Fig. 2, Fig. 3D), suggesting that it may act as a dominant negative by uncoupling the binding of Ndel1 to endogenous p600. Knockdown of Lis1 has been shown to disrupt the dynamics of astral MTs, their capture at the cell cortex, and to mislocalize Dynein (Yingling et al., 2008; Moon et al., 2014). Curiously, both of these defects can be rescued by overexpression of Ndel1, showing a redundancy and/or mutual dependency between Lis1 and Ndel1 (Yingling et al., 2008; Moon et al., 2014). Moreover, a direct and phosphorylation-dependent binding between Lis1 and Ndel1 is required for mitotic spindle orientation and aNP maintenance (Xie et al., 2013).

One possibility would be that p600^4480–5183^ regulates Ndel1 through direct interaction with the coiled-coil region and this interaction could alter the distribution and/or function of the Ndel1/Lis1/Dynein complex, thereby reducing their function(s) as stabilizers of the orientation of the spindle at the cell cortex. In support of this idea, we found that expression of p600^4480–5183^ alters the cytosolic/membrane localization of Ndel1, the Ndel1/Lis1 ratio so critical for the control of Dynein activity, as well as Dynein localization in HeLa cells (supplementary material Fig. S3). As Dynein function and localization are key for correct spindle orientation, our data provide a first evidence that p600 may regulate spindle orientation through Ndel1/Lis1/Dynein. Because these results were obtained in an artificial system (i.e. HeLa cells), further studies are required to substantiate these findings.

A complete understanding of the mechanism by which p600 and Ndel1 interact to control spindle orientation will also require us to localize the functional domains of p600 with greater accuracy and to test the above hypothesis biochemically and in neural progenitors. The dearth of data on the localization of functional domains and post-translational modifications within p600 denies us the opportunity to focus on likely areas. This absence of probable targets, combined with the humongous size of p600 and the variable solubility of the relevant C-terminal regions, make the full characterization of the p600/Ndel1 interaction by domain-mapping or mutagenesis an unusually daunting task, and drive it out of the scope of this paper. Based however on the direct p600/Ndel1 interaction, the dominant-negative effect of the Ndel1-binding p600^4480–5183^, and the close phenotypic similarity between *p600* conditional null, *Ndel1* and *Lis1* knockout mice, we propose that p600 controls spindle orientation of mitotic aNPs through the well-characterized actions of the Ndel1/Lis1/Dynein complex.

### What causes microcephaly in p600^SC−/−^ mice?

Microcephaly or “smallness of the brain” is typically the result of a substantial depletion of NPs caused by apoptosis, autophagy, and/or faster terminal differentiation of these NPs, resulting in overall decreased production of neurons. p600 is a multifunctional protein with key roles in basic cellular processes such as protein degradation (ubiquitin/proteasome-mediated degradation or autophagy), cell adhesion, cell survival and anoikis (a form of apoptosis induced by cell detachment). Thus, microcephaly in p600^SC−/−^ animals could be due to alterations in one or several of these cellular processes that, perhaps, could be inter-related. For example, p600 (also known as UBR4) belongs to the ‘UBR box motif’-containing family of proteins and acts as N-recognin in the N-end rule proteolytic pathway of the ubiquitin system. Like other N-recognins, p600 binds to a destabilizing N-terminal residue of a substrate protein and participates in the formation of a substrate-linked multiubiquitin chain, leading eventually to the degradation of the substrate (Tasaki et al., 2005). Interestingly, the ubiquitin–proteasome system (UPS) plays key roles during neurodevelopment (including neurogenesis), and a number of UPS-associated protein mutations have been identified in neurodevelopmental disorders (Naujokat, 2009). Furthermore, double KO UBR1^−/−^UBR2^−/−^ embryos die at midgestation, with defects in neurogenesis (Naujokat, 2009). Taken together, these results suggest that the degradation function of p600 may be compromised in p600^SC−/−^ mice and may contribute to altered neurogenesis and microcephaly.

Randomization of the spindle orientation in aNPs has been associated with apoptosis an accelerated terminal neuronal differentiation (Yingling et al., 2008), respectively. Previous mouse models with altered neural progenitor maintenance and survival develop smaller brain (Chae and Walsh, 2007). Thus, microcephaly observed in p600^SC−/−^ mice may be due deregulation of spindle orientation followed by apoptosis and accelerated neuronal differentiation of aNPs. In support of this hypothesis, our preliminary data indicate an increased signal for cleaved (active) caspase-3, a marker for apoptosis, in the brain of E12.5 p600^SC−/−^ brain populated with NESCs, but not limited to the ventricular zone (data not shown). Furthermore, aNPs in E15 neocortices (mostly RGCs) depleted of p600 or expressing the Ndel1-binding p600 fragment exhibit tilted spindle (Fig. 2B, Fig. 3D) and premature neuronal differentiation, as evidenced by the decreased number of Pax6-positive aNPs and increased number of Tbr2-positive basal progenitors destined to become neurons (Fig. 4). At E14 spindle orientation of Pax6-positive p600 shRNA or p600^4480–5183^-electroporated aNPs is randomized (Fig. 5). The randomization is nearly indistinguishable from the randomization in the total population of aNPs electroporated at E13 and analyzed at E15 (Fig. 2B, Fig. 3D), indicating that the analysis at E15 was unlikely done on BPs. Our data also constitute circumstantial evidence linking spindle randomization to the premature differentiation and subsequent depletion of Pax6-positive aNPs (Fig. 1C), decreased Tuj-1 production and microcephaly in p600^SC−/−^ mice (Fig. 6). Note that the decreased production of newly born neurons and randomized spindle orientation observed in our p600^SC−/−^ mice are readily recreated by electroporation of p600 RNAis. Hence while p600 is required in other tissues (Nakaya et al., 2013; Tasaki et al., 2013), the phenotype we have observed in aNPs cannot simply be epiphenomenal to a generalized growth defect. In sum, microcephaly in p600^SC−/−^ mice may be caused by a combination of mechanisms including tilting of the mitotic spindle and/or loss of the protein degradation function or other functions of p600.

### Final remarks

Previous studies on p600's roles in the brain dealt with later populations of migrating neurons (Shim et al., 2008), and with active mature neurons (Belzil et al., 2013). By contrast, the phenotype of p600^SC−/−^ mice originates much earlier in neural progenitors and is associated with decreased Tuj-1 production. Because of the drastically different circumstances and behavior of the cell types analyzed by these studies, the mechanism described herein is distinct from those described in previous works (Shim et al., 2008; Belzil et al., 2013). In this study, the requirement for p600 in spindle orientation was ascribed mostly to NESCs and RGCs, based on our analysis of aNPs in the ventricular zone between E12.5 and E15. Because the p600^SC−/−^ genotype is lethal as early as E9.5, completely lethal by E14.5, and because of the relative variability of this lethality (Nakaya et al., 2013; Tasaki et al., 2013), culturing p600^SC−/−^ progenitors was impossible and our experimental manipulations were restricted to the early aNPs, NESCs and RGCs. Based on the important role of p600 in these progenitors, one can justly hypothesize that p600 is also important for the biology of other NPs such as the monopolar (outer-subventricular-zone precursors, short neural precursors) and non-polar progenitors (BPs, inner subventricular zone progenitors) (Fietz and Huttner, 2011; Hansen et al., 2010). p600 may also play a role in the generation of neurons in the mature brain. Further study of p600 in NP populations will provide a better understanding of the roles of p600 in cell fate determination and neurogenesis in the developing and adult brain.

## Supplementary Material

Supplementary Material
