# Supplementary material for: p600 regulates spindle orientation in apical neural progenitors and contributes to neurogenesis in the developing neocortex
Source: Biol Open. 2014 May 8;3(6):475–85. doi: 10.1242/bio.20147807 (PMC4058081; doi:10.1242/bio.20147807)
Supplement: Supplementary Material [file supp_3_6_475__index.html]

p600 regulates spindle orientation in apical neural progenitors and contributes to neurogenesis in the developing neocortex — Supplementary Material 

# p600 regulates spindle orientation in apical neural progenitors and contributes to neurogenesis in the developing neocortex

## bio.20147807 Supplementary Material

**Files in this Data Supplement:**

- Supplementary Material - Camille Belzil et al. doi: 10.1242/bio.20147807
