# Supplementary material for: p600 regulates spindle orientation in apical neural progenitors and contributes to neurogenesis in the developing neocortex
Source: Biol Open. 2014 May 8;3(6):475–85. doi: 10.1242/bio.20147807 (PMC4058081; doi:10.1242/bio.20147807)
Supplement: Supplementary Material [file supp_bio.20147807_bio.20147807-s1.pdf]

Biology Open (2014) 000, 1–11 doi:10.1242/bio.20147807

Supplementary Material
Camille Belzil et al. doi: 10.1242/bio.20147807

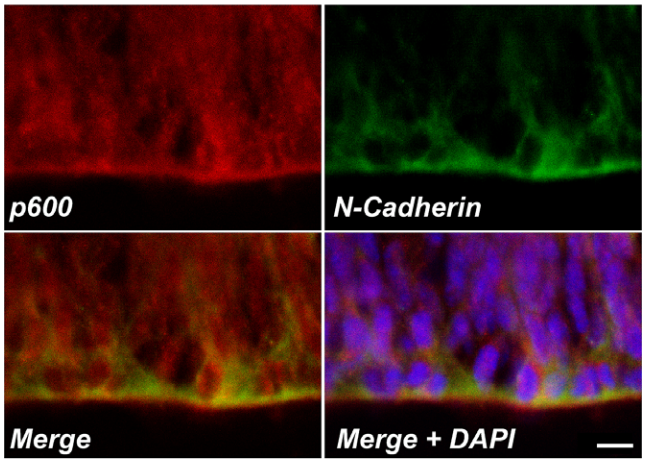

Fig. S1. p600 is expressed in apical neural progenitors of the neocortex at E12.5. p600 is expressed in aNPs of the ventricular zone in the neocortex at E12.5 as revealed by co-labeling with N-Cadherin antibody. Scale bar: 12 µm.

Biology Open (2014) 000, 1–11 doi:10.1242/bio.20147807

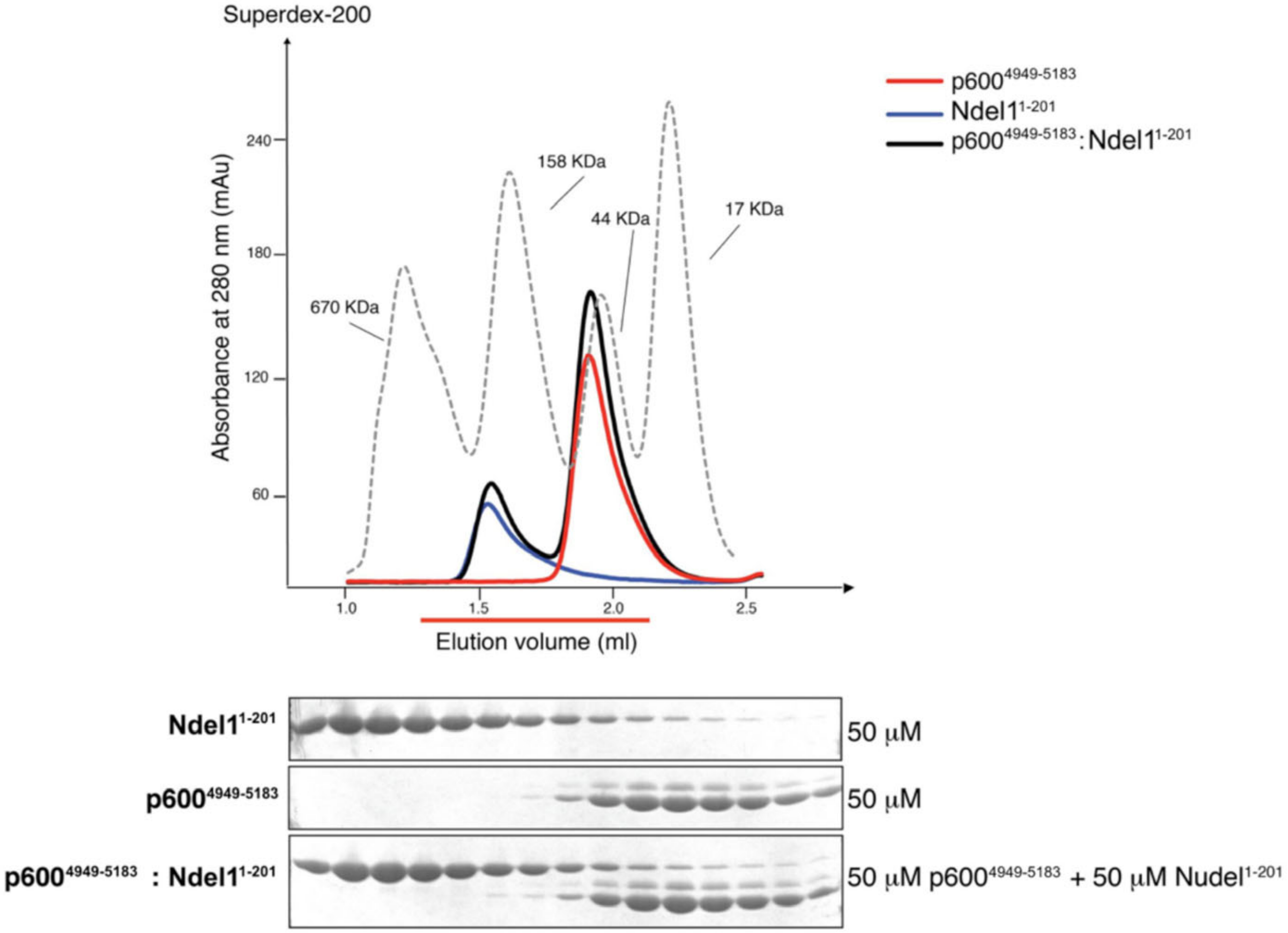

Fig. S2. The C-terminal portion of p600 fragment C (a.a. 4949–5183) does not bind Ndel1-cc. To identify the minimal region of p600 required for the association with Ndel1-cc, a construct of p600 encompassing the very C-terminal portion of fragment C (residues 4949–5183) was generated. Its ability to bind Ndel1$^{1\text{-}201}$ was assessed by size exclusion chromatography (SEC). p600 (4949–5183) elutes from SEC in a single peak (red trace), much later than Ndel1-cc (blue trace). When mixed at equimolar 50 μM concentration, p600 (4949–5183) and Ndel1-cc elute into two separate peaks corresponding to the two species in isolation (black trace). These results indicate that they do not form a complex in solution.

Biology Open

Biology Open (2014) 000, 1–11 doi:10.1242/bio.20147807

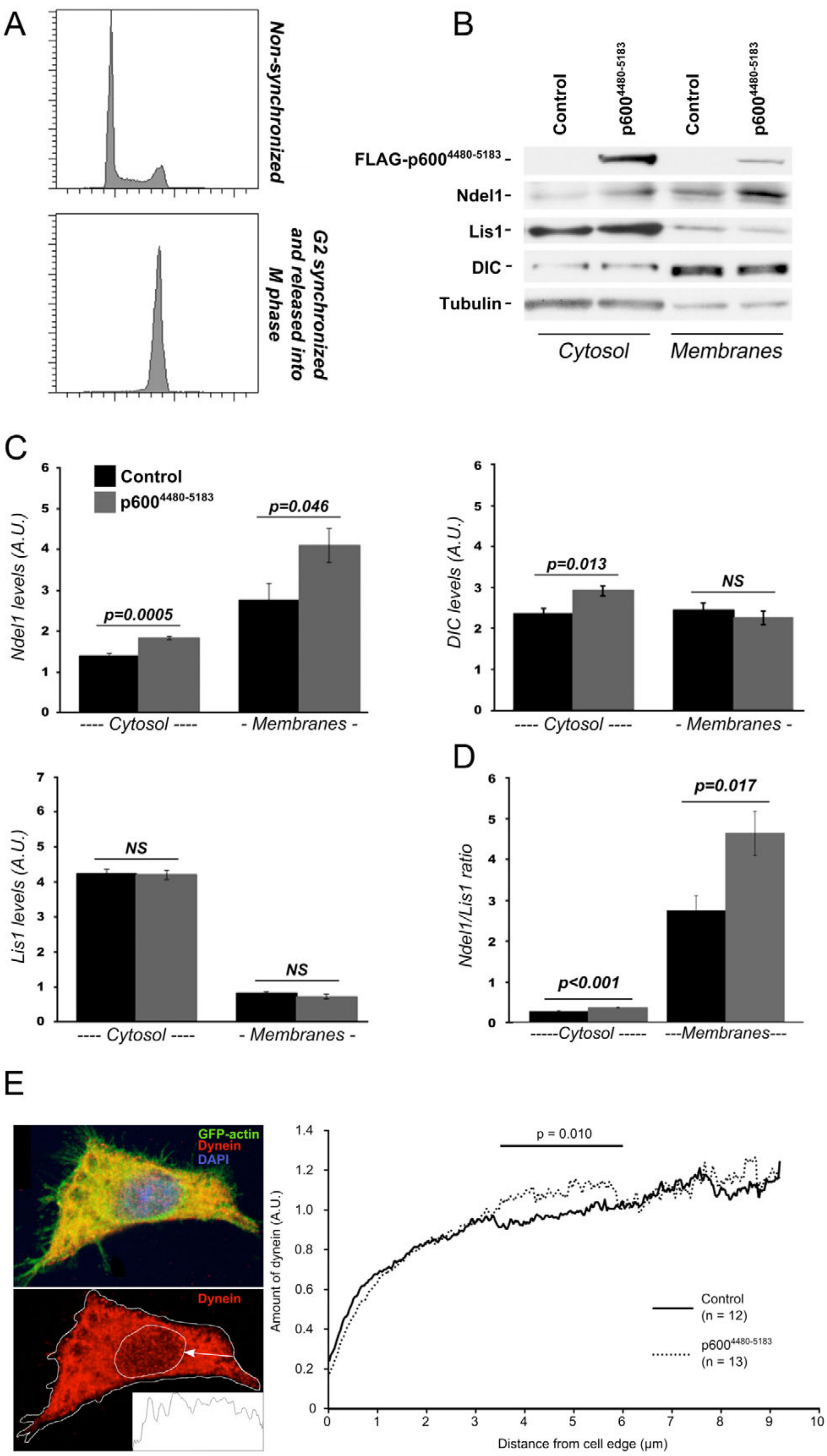

Fig. S3. Expression of $p600^{4480–5183}$ alters the levels of cytosolic Ndel1 and Dynein, the ratio of Ndel1/Lis1, and cytosolic Dynein distribution in HeLa cells. (A) FACS analysis showing the high synchronization of HeLa cells at the G2 phase. The G2 synchronization was followed with a 30 min release, enabling cells to enter mitosis. (B) Cytosolic and membrane distribution of Ndel1, Lis1, and Dynein in mitotic HeLa cells transfected with an empty vector or a construct encoding FLAG-tagged Ndel1-binding $p600^{4480–5183}$ (detected with an anti-FLAG antibody). In this type of fractionation, Ndel1 is more enriched in the membrane fraction whereas Lis1 is predominantly found in the cytosol. Dynein is distributed more evenly in both fractions. (C) $p600^{4480–5183}$ expression significantly enhanced the levels of Ndel1 (by 33%, $t_{10}=5.01$, $p=0.00053$) and Dynein intermediate chain (by 23%, $t_{10}=3.03$, $p=0.013$) but not of Lis1 ($t_{10}=0.128$, $p=0.90$) in the cytosolic fraction. No difference in the membrane localization of Dynein was found. Bar graphs showing the levels of Ndel1, Lis1 and Dynein intermediate chain (DIC) in the cytosolic and membrane fractions of mitotic HeLa cells expressing an empty vector or $p600^{4480–5183}$. A.U.: arbitrary units; NS: non-significant. Expression of $p600^{4480–5183}$ enhances the levels of Ndel1 in both fractions and the levels of DIC in the cytosolic fraction. (D) Heightened levels of Ndel1 in these compartments, along with unaltered levels of Lis1, leads to a significant shift (~34%) of the Ndel1/Lis1 ratio in both the cytosol and membrane fractions by 34% ($t_{10}=0.491$, $p=0.00061$) and 69% ($t_{10}=2.87$, $p=0.017$), respectively. The Ndel/Lis1 ratio is predictive of changes in Dynein distribution and function. (E) The confocal image shows an example of Dynein distribution in the cytosol. DAPI and GFP-actin transfection (top image) were used to delimit the nucleus and cell edge respectively (white traces in bottom image). A sample measurement of Dynein intensity in a line from the cell edge to the nucleus is shown. The Dynein distribution of each cell, representing 36 such measurements, was averaged to generate the plot on the right. Transfection with $p600^{4480–5183}$ caused an accumulation of Dynein between 3.5 and 6 µm from the cell edge ($F_{1,23}=7.78$, $p=0.010$).
